# Improving the Energy Storage of Supercapattery Devices through Electrolyte Optimization for Mg(NbAgS)_x_(SO_4_)_y_ Electrode Materials

**DOI:** 10.3390/molecules28124737

**Published:** 2023-06-13

**Authors:** Haseebul Hassan, Muhammad Waqas Iqbal, Sarah Alharthi, Mohammed A. Amin, Amir Muhammad Afzal, Jacek Ryl, Mohd Zahid Ansari

**Affiliations:** 1 Department of Physics, Riphah International University, Campus Lahore, Lahore 54000, Pakistan; ah0637603@gmail.com (H.H.); amir.afzal@riphah.edu.pl (A.M.A.); 2 Department of Chemistry, College of Science, Taif University, Taif P.O. Box 11099, Saudi Arabia; sarah.alharthi@tu.edu.sa (S.A.); mohamed@tu.edu.pl (M.A.A.); 3 Division of Electrochemistry and Surface Physical Chemistry, Faculty of Applied Physics and Mathematics, Gdańsk University of Technology, Narutowicza 11/12, 80-233 Gdańsk, Poland; 4 School of Materials Science and Engineering, Yeungnam University, 280 Daehak-Ro, Gyeongsan 38541, Republic of Korea

**Keywords:** safe electrolytes, electrolyte optimization, binder-free electrode material, ternary metallic sulfides, supercapattery devices

## Abstract

Electrolytes are one of the most influential aspects determining the efficiency of electrochemical supercapacitors. Therefore, in this paper, we investigate the effect of introducing co-solvents of ester into ethylene carbonate (EC). The use of ester co-solvents in ethylene carbonate (EC) as an electrolyte for supercapacitors improves conductivity, electrochemical properties, and stability, allowing greater energy storage capacity and increased device durability. We synthesized extremely thin nanosheets of niobium silver sulfide using a hydrothermal process and mixed them with magnesium sulfate in different wt% ratios to produce Mg(NbAgS)_x_)(SO_4_)_y_. The synergistic effect of MgSO_4_ and NbS_2_ increased the storage capacity and energy density of the supercapattery. Multivalent ion storage in Mg(NbAgS)_x_(SO_4_)_y_ enables the storage of a number of ions. The Mg(NbAgS)_x_)(SO_4_)_y_ was directly deposited on a nickel foam substrate using a simple and innovative electrodeposition approach. The synthesized silver Mg(NbAgS)_x_)(SO_4_)_y_ provided a maximum specific capacity of 2087 C/g at 2.0 A/g current density because of its substantial electrochemically active surface area and linked nanosheet channels which aid in ion transportation. The supercapattery was designed with Mg(NbAgS)_x_)(SO_4_)_y_ and activated carbon (AC) achieved a high energy density of 79 Wh/kg in addition to its high power density of 420 W/kg. The supercapattery (Mg(NbAgS)_x_)(SO_4_)_y_//AC) was subjected to 15,000 consecutive cycles. The Coulombic efficiency of the device was 81% after 15,000 consecutive cycles while retaining a 78% capacity retention. This study reveals that the use of this novel electrode material (Mg(NbAgS)_x_(SO_4_)_y_) in ester-based electrolytes has great potential in supercapattery applications.

## 1. Introduction

With a growing need for environmentally friendly and stable sources of energy, the requirement for effective energy storage devices has expanded dramatically [1,2,3,4]. Supercapattery devices have been developed as a viable alternative for high-power and high-energy applications, combining the features of supercapacitors (SCs) and batteries (LIBs) [5,6,7]. The wider application of SCs is limited by energy density and life cycle restrictions [8]. To address these issues, major research efforts have been directed toward developing better electrode materials and optimizing electrolyte composition [9,10,11].

The significance of electrolyte selection in capacitors cannot be overestimated [12,13,14]. Capacitors hold energy as an electric charge, and the quantity of energy kept corresponds to the square of the voltage [15,16]. Organic electrolyte SCs appear as an appealing choice in this regard because of their broader potential window when compared to aqueous electrolyte SCs [17,18]. Aqueous electrolytes have a narrow potential window, frequently constituted of water-based solutions. This reduces the range of voltages that may be applied over the capacitor, limiting the energy storage capacity [18,19]. Organic electrolytes, in contrast, provide a larger potential window, allowing for greater voltages to be supplied across the SCs [20,21]. This increased voltage range allows for the buildup of higher quantities of electric charge, resulting in improved energy storage capacities. Organic electrolyte SCs have a wider potential window due to the intrinsic electrochemical resilience of organic solvents, which allows them to sustain greater voltages without electrolyte degradation [22,23,24,25]. Different solvents were examined in this context including methoxy acetonitrile [26], g-butyrolactone [27], sulfolane [28], and methoxy propionitrile [29]. The solvents with flash points greater than 30 °C, such as methoxy propionitrile and methoxy acetonitrile, have greater electrical conductivity. Because of the greater viscosity of sulfolane and g-butyrolactone, the conductivity values are lower. However, this may be countered by high electrochemical strength, allowing the working voltage of the SCs to be increased [29,30,31]. S. Ike et al. [32] used EC electrolyte with the addition of butylene carbonate and propylene carbonate solvents which improves the electrochemical performance of SCs.

Silver sulfide adds to the capacitance and energy storage properties of the composites. Because of its high specific capacitance and outstanding redox behavior, Ag_2_S enables reversible charge storage and overall system stability. Niobium insertion into the composite provides benefits such as increased electrical conductivity and a large specific surface area, which improves efficient charge transfer and promotes favorable electrode–electrolyte interactions. Niobium also has strong chemical stability and a wide electrochemical potential window, allowing it to resist higher voltages without degrading [33,34]. The conductivity, capacitance, and stability of the composite were optimized by altering the weight ratio of Nb to Ag_2_S, consequently enhancing the electrochemical performance in terms of energy storage density, cycle stability, and charge–discharge efficiency [35]. Hassan et al. [36] synthesized NiAg_2_S using a hydrothermal approach which delivers an energy density of 28.97 Wh/kg. The incorporation of MgSO_4_ into metal sulfides has yielded good results in terms of increasing the electrochemical characteristics of SCs. MgSO_4_ functions as an electrolyte addition, improving the overall stability and conductivity of the electrode material. MgSO_4_ insertion in metal sulfides has been shown to boost specific capacitance, improve cycle stability, and increase the overall energy density of the SCs [37,38]. Mg(NbAgS)_x_(SO_4_)_y_ combines the characteristics of MgSO_4_ with NbAgS, resulting in synergistic effects that increase the total energy density of the electrode materials. The combination improves electrochemical performance and increases ion storage capacity, resulting in better energy density in supercapacitors. Mg(NbAgS)_x_(SO_4_)_y_ enables the storage of multivalent ions that are stable in the electrode material, such as Mg^2+^ and Nb^4+^. When contrasted with single-ion storage in materials such as MgSO_4_ or NbAgS, the capacity to store multivalent ions considerably boosts energy density since more charge may be stored per unit volume or mass. The study of Mg(NbAgS)_x_(SO4)_y_ as an SC electrode has great promise for developing energy storage technology. SCs are well known for their outstanding power density, quick charging and discharging rates, and extended life span. Researchers hope to discover more about Mg(NbAgS)_x_(SO4)_y_ as an electrode and evaluate its potential for SC applications.

Finally, this work represented the synthesis and characterization of Mg(NbAgS)_x_(SO_4_)_y_ binary composites for SC applications. We were successful at producing innovative composite materials with customized compositions by mixing magnesium sulfate (MgSO_4_) and niobium silver sulfide (NbAgS) in regulated quantities. The hydrothermal synthesis approach allows for the production of well-defined structures as well as a homogeneous distribution of elements within the composite. The inclusion of Mg, Nb, Ag, S, and SO_4_ in the composite material provides unique benefits such as better electrochemical performance and energy storage capabilities. This work focuses on solvent modification in electrolytes for SCs. The ester when used as a co-solvent causes a balance among electrochemical performance across a broad range of temperatures and safety considerations. In this work, two different salts, tetraethylammonium tetrafluoroborate (TEABF_4_) and spiro-(1,10)-bipyrrolidinium tetrafluoroborate (SBPBF_4_), were investigated.

## 2. Experimental Section

### 2.1. Materials

Two sources provided the essential chemical components for this investigation. The MERC supplied the requisite volumes of magnesium chloride (MgCl_2_·3H_2_O), silver chloride (AgCl·3H_2_O), sodium sulfide (Na_2_S·9H_2_O), and NbCl_2_, all of which were of excellent purity and ensured the results’ correctness. N-poly vinylidene fluoride (PVDF), potassium source (KOH), carbon black, activated carbon, and Sigma Aldrich supplied all N-Methyl-2-pyrrolidone. These materials were of exceptional grade and were essential to the composites’ effective synthesis. ALS Co., Ltd. also supplied the nickel foam (NF), counter electrode (pt/wire), and reference electrode (Hg/HgO). Obtaining high-quality materials from reliable vendors was crucial to ensure the precision and dependability of the trial outcomes.

### 2.2. Materials Synthesis

Mg(NbAgS)_x_(SO_4_)_y_ composites (MNAS-1, MNAS-2, MNAS-3, and MNAS-4) were synthesized using the hydrothermal technique. This method was chosen due to its simplicity and low cost. When compared to alternative synthesis processes, such as chemical co-precipitation or sonochemical procedures, hydrothermal synthesis produced materials with greater degrees of crystallinity. Depending on the materials being synthesized, the hydrothermal technique can be used at temperatures ranging from 140 to 300 °C [39]. The synthesis of MNAS-1 to MNAS-4 involves the use of certain ratios of MgSO_4_ and NbAgS. NbAgS was synthesized using the hydrothermal method. To begin, two solutions were prepared. First, 0.8 M niobium chloride (NbCl_2_) and 0.8 M silver chloride (AgCl·3H_2_O) were prepared in 20 mL of DIW. Solution 2, containing 1.6M thiourea dissolved in 20 mL DI water, was prepared. The solution was subsequently combined with the niobium chloride and silver chloride (AgCl·3H_2_O) solution and stirred for 25 min. The resulting mixture was transferred to an autoclave and heated to a temperature of 180 °C for 12 h.

(1) $$NbCl_{2}+AgCl+CS(NH_{2}{)}_{2}+2NaOH\overset{H_{2}O}{\to }NbAgS+2NaCl+Cl^{-}+CO_{2}+2NH_{3}$$

Similarly, we prepared MgSO_4_ by dissolving 0.8 M of magnesium chloride (MgCl_2_·3H_2_O) and 1.6 M of thiourea in 500 mL of DIW. We adjusted the pH of the solution to 10 by introducing a 6 M NaOH solution. We followed the previously described steps for the synthesis of MgSO_4_

(2) $$MgCl_{2}+CS(NH_{2}{)}_{2}+2H_{2}O+2NaOH\overset{H_{2}O}{\to }MgSO_{4}+2NH_{3}+2NaCl+CO_{2}+4H_{2}$$

We combined MgSO_4_ and NbAgS in the appropriate ratios using the hydrothermal process to produce four composites, namely MNAS-1, MNAS-2, MNAS-3, and MNAS-4. A synthesis of MNAS-1 involved utilizing two different solutions. The first solution contained 0.2 M of NbAgS dissolved in 20 mL of DIW, while the second solution consisted of 0.8 M of MgSO_4_ dissolved in 20 mL of DIW. Solution 2 underwent continuous mixing with solution 1 for 25 min. Afterward, the mixture was placed in a stainless steel autoclave and heated at 160 °C for 8 to 10 h. To eliminate unwanted particles, the resulting product underwent a cooling process to reach room temperature, followed by a thorough cleaning using ethanol and DIW.

**Figure 1:**
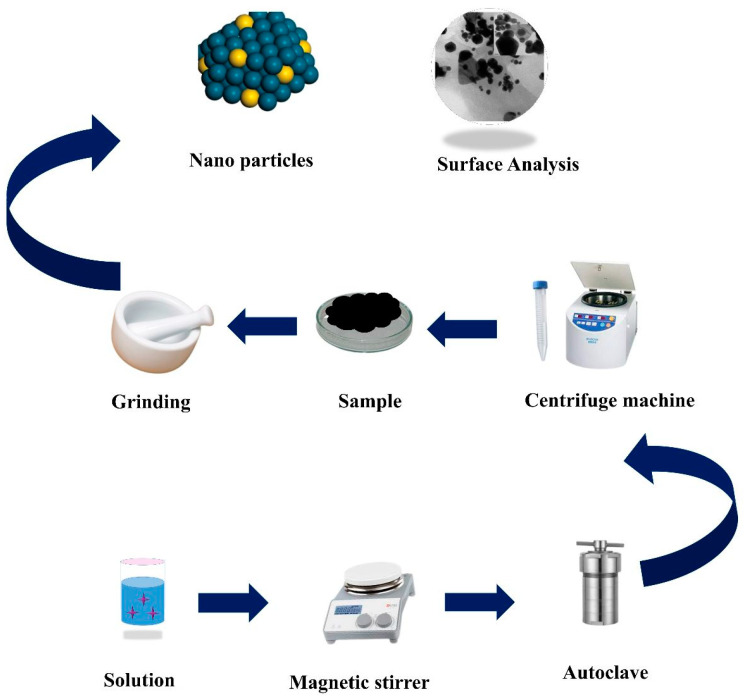
Systematic representation for the synthesis of Mg(NbAgS)_x_(SO_4_)_y_ through the hydrothermal process.

Afterward, the product was dried for 3–4 h at 70 °C before collecting it as MNAS-1. To synthesize MNAS-2, Solution 1 contained 0.5M of NbAgS, while Solution 2 contained 0.8M of MgSO_4_. MNAS-3 required one mole of NbAgS and one mole of MgSO_4_, whereas MNAS-4 required 0.8 M of NbAgS and 0.2 M of MgSO_4_ in Solutions 1 and 2, respectively. Table S1 represented the composition of materials in MNAS-1 to MNAS-4 composites. Figure 1 presents a systematic illstration of the hydrothermal technique used to synthesize these composites (MNAS-1 to MNAS-4).

### 2.3. Characterization

Rigaku Ultima III X-ray diffractometer was used to determine the crystallinity and phase uniformity of the active materials. The surface properties were studied using scanning electron microscopy (Hitachi-S-4800, Hitachi High-Technologies, Tokyo, Japan). X-ray photoelectron spectroscopy (Thermo, Pittsburgh-XPS, Thermo Fisher Scientific, Waltham, MA, USA) was applied to analyze the chemical composition of the synthesized compounds. The surface area of the synthesized materials was investigated using Quantachrome Autosorb AS-6B. In addition, the electrochemical activity for all materials was studied using the Corrtest CS300 potentiostat.

### 2.4. Thermal Calculations

The thermal study of all synthesized compounds was carried out using a DSC 2920 CE analyzer. The samples, each weighing 5 mg and carefully packed in aluminum pans inside a controlled atmosphere, were placed inside a glove box. Using a perfectly controlled heating technique, the samples were exposed to temperature variation spanning from 100 °C to 40 °C, followed by a cooling phase from 40 °C to a lower limit of −100 °C, both at a constant rate of 5 °C per minute. As a result, the critical melting temperature (Tm) was supplied by the apex of the melting peak, while the significant crystallization temperature (Tc) was provided by the pinnacle of the crystallization peak.

### 2.5. Viscosity Calculations

The capillary viscometer was used to determine the viscosity of the material under consideration. To ensure accuracy, 10 measurements were taken, and the kinematic viscosity was calculated by taking the average. These readings were within a stunning 1% margin of error. The dynamic viscosities, on the other hand, were calculated using the following formula: η=Kρt. In this equation, *K* reflects the viscometer’s constant (0.01032 mm^2^/s^2^), whereas ρ represents the density of the electrolyte at 40 °C (given in g/cm^3^). Finally, the variable *t* represents the time it took the electrolyte to transit the capillary (in seconds).

### 2.6. Conductivity Analysis

The conductivities were determined with the help of an HP 4192A Impedance Analyzer. This investigation included a frequency range of 5 Hz to 13 MHz. We used a glass cell with two platinum electrodes uniformly spaced apart, carefully arranging the samples to experiment. The experiments took place in an argon atmosphere, covering temperatures ranging from −30 to 70 °C. A one-hour temperature equilibration interval preceded each measurement to assure stability and precision. To check measurement reliability, the conductivity of a particular electrolyte was measured twice using two different conductivity cells. The predicted uncertainty in the conductivity measurements is 0.3 mS/cm. A solution with a concentration of 0.1 M was used to calculate the cell parameter.

### 2.7. Electrochemical Testing

Electrochemical tests (CV, GCD, and EIS) were recorded precisely within a glove box in a controlled setting at ambient temperature. The counter electrode was a platinum wire, while the working electrode was made of Mg(NbAgS)_x_(SO_4_)_y_ composites. A scan rate of 3–50 mV/s was used to achieve precise readings. An Ag wire was submerged in a solution of 10 mM AgNO_3_ in acetonitrile supplemented with 0.1 M tetrabutylammonium BF_4_ as the reference electrode. A value of 0.542 V was applied to the recorded potential to convert it to the NHE (Normal Hydrogen Electrode) scale. By measuring the molar concentration of medium polarity solvents in ACN (acetonitrile) supplemented with 1 M TEABF_4_ electrolytes, the electrochemical stability of the solvents was evaluated [40].

The electrode composition was 10% PVDF (Polyvinylidene Fluoride) and 10% conductive additives with an active material loading of 2.94 mg/cm^2^. Extensive previous research has shown that these compounds have little to no effect on the stability of the electrolyte. The electrodes were kept in a glove box after drying at 100 °C under vacuum conditions. Cyclic voltammetry was performed in three- and two-electrode setups in which a cellulose-based splitter was used to separate electrodes with porosities greater than 70% and a thickness of 25 mm. Galvanostatic charge/discharge (GCD) was performed at 1–3 A/g and operating voltages ranging from 0 to 3 V. During the galvanostatic charge/discharge investigation, the time constant was computed by examining the “IR” reduction observed during the starting region of the discharge curve.

### 2.8. NMR Calculations

NMR studies were meticulously carried out using a 9.4 T Bruker Avance 400 NMR spectrometer outfitted with a Bruker 5 mm hybrid broadband/1H-19F sensor. This probe had a *z*-axis and a temperature controller, with a remarkable 0.2 °C stability and precision. The proton (1H) and fluorine (19F) nuclei’s NMR resonance frequencies were 400.13 MHz and 376.50 MHz, respectively. The pulsed field variation triggered echo and LED sequences, with two spoil gradients (PFG NMR), was used to perform self-diffusion experiments. The amplitude of the pulsed field gradient was adjusted from 0 to 40 G/cm. The diffusion time (*D*), which represents the time between two pulses, was held constant at 100 ms, and the duration of the gradient pulse (*d*) was varied between 3 and 18 milliseconds, depending on the diffusion coefficient of the mobile species under study. The decrease in the spin echo frequency was detected using this technology across a wide range extending at least two decades. This method yielded a high degree of accuracy (5%) when calculating the self-diffusion coefficient values. These values were calculated using the formula $\ln\left(\frac{I}{I_{o}}\right)=-Dg^{2}\gamma^{2}\delta^{2}\left(∆-\frac{\delta }{3}\right)$, where *g* indicates the amplitude of the two gradient pulses and *g* is the gyromagnetic ratio of the individual nucleus under consideration. *I* and *I_o_* indicate the signal area acquired with and without gradient pulses, respectively. This equation incorporates the diffusion coefficient (*D*), gradient pulse duration (*d*), and diffusion time (*D*), allowing for the correct estimation of self-diffusion coefficients.

## 3. Results and Discussion

### 3.1. Structural Analysis

**Figure 2:**
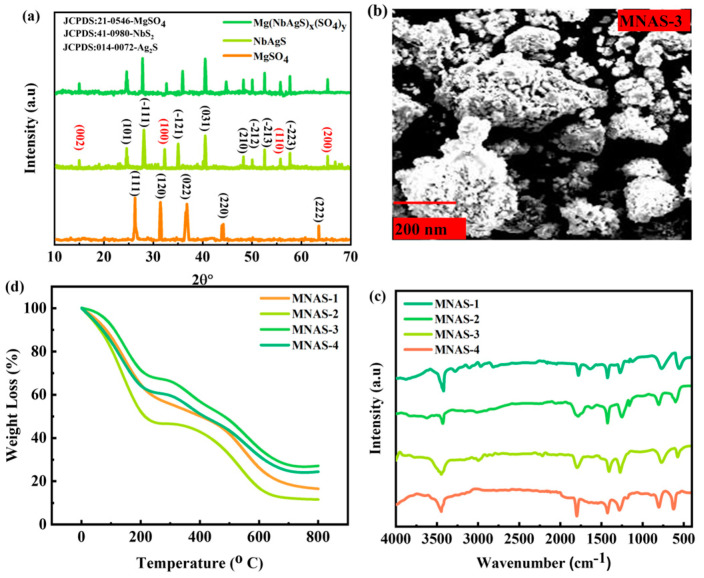
(**a**) XRD representation for MgSO_4_, NbAgS, and Mg(NbAgS)_x_(SO_4_)_y_. (**b**) SEM image for the best Mg(NbAgS)_x_(SO_4_)_y_ composite (MNAS-3). (**c**) TGA analysis of the four composites of Mg(NbAgS)_x_(SO_4_)_y_ (MNAS-1–MNAS-4). (**d**) FTIR spectrum for four composites of Mg(NbAgS)_x_(SO_4_)_y_ (MNAS-1–MNAS-4).

The X-ray diffraction (XRD) technique was used to study the structural properties of the synthesized nanomaterials. Figure 2a shows the XRD diffraction pattern for the MgSO_4_, NbAgS, and Mg(NbAgS)_x_(SO_4_)_y_ composites (MNAS-3). The XRD pattern for MNAS-1 revealed peaks that were consistent with JCPDS:21-0546-MgSO_4_ [41], JCPDS: 41-0980-NbS_2_ [42], and JCPDF-014-0072-Ag_2_S [43], showing that the composite was made up of hexagonal MgSO_4_, NbS_2_, and Ag_2_S composites. The diffraction spikes for MgSO_4_ appeared at 2θ = 26.1°, 31.8°, 36.5°, 44°, and 63.7° belonging to the diffraction planes of (111), (120), (022), (220), and (222). The diffraction spikes for Ag_2_S appeared at 2θ = 24.8°, 28°, 34.7°, 40°, 48.3°, 50.2°, 52.6°, and 58° belonging to the diffraction planes of (101), (−111), (−121), (031), (210), (−212), (−213) and (−223). Similarly, the diffraction peaks for NbS_2_ appeared at 2θ = 15.2°, 32.3°, 55°, and 64.8° belonging to the diffraction planes of (002), (100), (110), and (200). The XRD pattern for MNAS-2, MNAS-3, and MNAS-4, on the other hand also revealed the existence of MgSO_4_ and NbAgS. However, the intensities of the XRD peaks vary with concentration. Scherer’s formulas were used to calculate the crystallite size for all combinations [44].

(3) $$D=\frac{K\lambda }{\beta \cos\theta }$$

This equation yielded grain sizes of 0.49, 057, 0.73, and 0.64 nm for MNAS-1, MNAS-2, MNAS-3, and MNAS-4, respectively.

### 3.2. Surface Analysis

In this work, the surface morphology of the synthesized Mg(NbAgS)_x_(SO_4_)_y_ composites (MNAS-3) was examined using a scanning electron microscope (SEM), as indicated in Figure 2b. The SEM images revealed a highly porous and linked network structure in the synthesized composites. It can be seen that as the value of ‘x’ grows, so does the surface morphology of the composites. MNAS-3, for example, has a homogeneous and spherical morphology. The SEM analysis revealed important information on the surface morphology and microstructure of the synthesized composites, which will be beneficial in developing high-performance electrode materials for energy storage applications.

### 3.3. FTIR Analysis

The chemical bonds in the synthesized Mg(NbAgS)_x_(SO_4_)_y_ composites were characterized using Fourier-transform infrared spectroscopy (Figure 2c). The FTIR spectra of the four composites were collected in the 400–4000 cm^−1^ region. MNAS-1 exhibited a large absorption peak at roughly 3420 cm^−1^, which corresponds to the stretching vibrations of −OH groups of water molecules and the −NH groups of thiourea [45]. The peaks recorded at 1240 and 1410 cm^−1^ were attributed to SO_4_^2−^ ion stretching vibrations [46]. The peaks at 670 cm^−1^ and 735 cm^−1^ were found to be connected to the stretching vibrations of Mg-S and Nb-S bonds, respectively [42,47]. The MNAS-2, MNAS-3, and MNAS-4 FTIR spectra showed identical peak patterns, confirming the presence of the same functional groups as MNAS-1. The findings indicate that the synthesized composites have the appropriate chemical linkages, which are important for their electrochemical capabilities.

### 3.4. TGA Analysis

The thermal behavior of the four Mg(NbAgS)_x_(SO_4_)_y_ composites was investigated using thermogravimetric analysis (TGA), as indicated in Figure 2d. TGA is a strong analytical method that analyses the weight change in a sample as a function of temperature. The TGA study of the synthesized composites was carried out in a nitrogen gas flow from room temperature to 800 °C at a heating rate of 10 °C/min. TGA data showed each of the weight loss that occurred in the composites followed a similar pattern. Initially, the decomposition of water that had been absorbed and volatile pollutants in the sample resulted in a modest weight loss. Following that, significant weight loss occurred in the range of temperatures of 300–500 °C, and was ascribed to the breakdown of organic compounds such as thiourea and polyvinylidene fluoride [48]. In addition, the breakdown of MgSO_4_ aids in weight loss. Finally, at temperatures exceeding 600 °C, a slight weight loss was observed, which was attributed to the oxidation of remaining organic wastes. At a decomposition temperature of 550 °C, MNAS-3 exhibited the best thermal endurance as compared to the other four composites. This is because MNAS-3 has an equal amount of NbAgS and MgSO_4_, which boosts thermal endurance.

### 3.5. XPS Analysis

**Figure 3:**
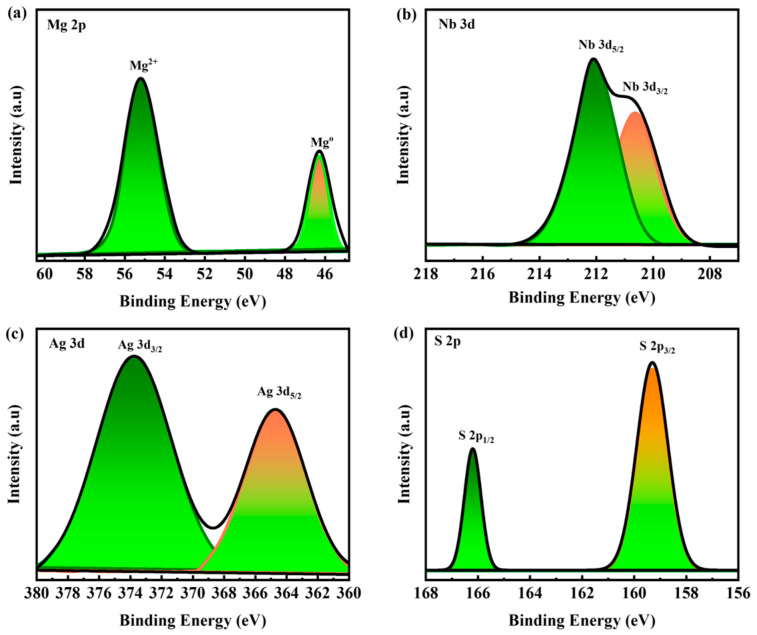
(**a**–**d**) XPS spectrum for Mg 2p, Nb 3d, Ag 3d, and S 2p, respectively.

X-ray photoelectron spectroscopy (XPS) was used to study the chemical states and elemental compositions of the Mg(NbAgS)_x_(SO_4_)_y_. Some other functional compounds, such as sulfates and thiourea, were also detected in the XPS spectra, indicating their significance in the formation of the composites. Deconvoluting the XPS spectra of the MNAS-3 composite yielded the element binding energies (Figure 3). The binding energy of Mg 2p is 55.7 and 46.4 eV [49]. The binding energy of Nb 3d_5/2_ and Nb 3d_3/2_ is 212.3 and 210 eV [50]. The binding energy of Ag 3d_5/2_ and Ag_3/2_ is 364.3 and 374 eV [51]. The binding energy of S 2p_3/2_ and S 2p_1/2_ in NbAgS is found to be 158.5 and 166 eV [52]. The XPS spectrum indicated that the Mg 2p_3/2_ peak was largely occupied by Mg^2+^ species, which was consistent with the formation of MgSO_4_. Nb^5+^ species comprised the Nb 3d_5/2_ peak, which was consistent with NbAgS production. The Ag 3d_5/2_ peak was largely occupied by Ag^+^ species, indicating that Ag_2_S was produced. SO_4_^2−^ species dominating the SO_4_ 2p_3/2_ peak, which was consistent with MgSO_4_ generation.

### 3.6. BET Analysis

**Figure 4:**
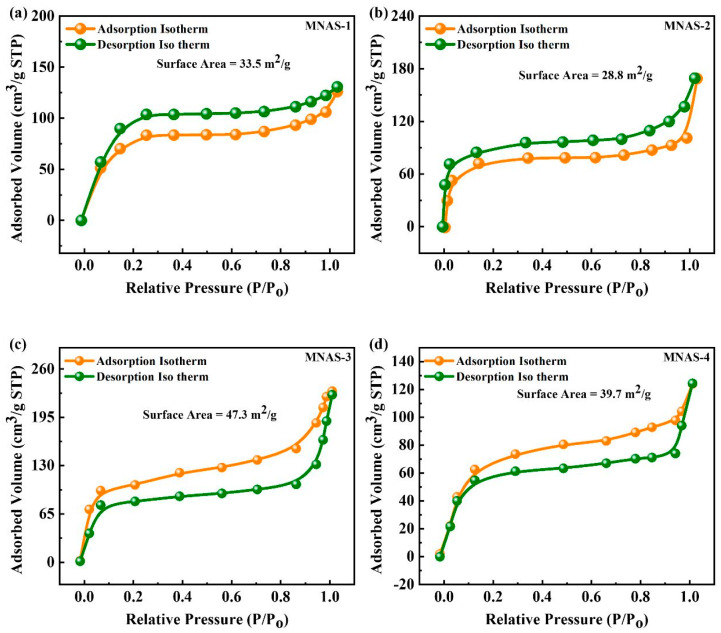
(**a**–**d**) BET isotherm for MNAS-1 to MNAS-4.

The surface area (SA) of four different Mg(NbAgS)_x_(SO_4_)_y_ compounds (MNAS-1–MNAS-4) was examined using the Brunauer–Emmett–Teller (BET) technique, as indicated in Figure 4. The BET technique is widely used to compute the specific SA of porous substances. It depends on the adsorption of gas particles onto the surfaces of materials and the calculation of SSA using the adsorption isotherm. MNAS-1 to MNAS-4 show a Type IV curve with an H-1 hysteresis cycle, which is typical of mesoporous substances with cylindrical or slit-shaped porosity [53]. The mesoporous structure of Mg(NbAgS)_x_(SO_4_)_y_ was confirmed by the adsorption of molecules at reduced pressure. MNAS-3 exhibited the greatest SSA of 47.3 m^2^/g, the smallest pore volume of 0.044 cm^3^/g, and the smallest pore size of 15 nm. The mesopores have a uniform size distribution, allowing for efficient gas adsorption and desorption. Table 1 represented the computed parameters for MNAS-1, MNAS-2, MNAS-3, and MNAS-4 through BET isotherms. MNAS-3 is capable of being utilized as an electrode material for supercapacitors due to its high surface area and large pore volume. The high surface area and porosity of MNAS-3 can provide a large region for the adsorption of electrolyte ions, which can increase the capacitance of the device. The increased pore capacity can facilitate electrolyte infiltration and ion transport, resulting in higher performance. As a result, MNAS-3 has a lot of potential in supercapacitor applications.

## 4. Electrolytic Studies

Certain qualities, such as strong ionic conductivity, high electrochemical stability, and thermal stability, are required to satisfy the criteria of a supercapacitor electrolyte. At low temperatures, ethyl acetate (EA) solvent is often employed in LIBs technology along with carbonate solvents that improve the conductivity of the ions. Furthermore, when mixed with TEABF_4_, EA exhibits extraordinary electrochemical stability, ranging from 2.5 V in oxidation and −2.8 V in reduction against Ag/Ag^+^, which is most likely due to the reduction of the BF_4_ anion. However, EA has certain disadvantages, including strong volatility, poor polarity, and a high amount of flammability. Different ester-based solvents with nitrile, methoxy, or halogen as functional groups were investigated to resolve these restrictions without reducing viscosity. The physicochemical parameters of the solvents investigated are summarized in Table S2. The flash point of all solvents was greater than 20 °C, greatly outperforming EA in terms of safety. The addition of nitrile or methoxy polar groups causes an increase in the flash point and dielectric constant. The MCA has a flash point (Fp) of 43 °C and a dielectric constant (r) of 28. When evaluating methylmethoxyacetate (MMOA) (Fp = 35 °C, r = 0.82) to propyl acetate (Fp = 25 °C, r = 4.4), an analogous trend was seen. The flash point of chloromethyl butyrate (ClMB) at 55 °C with a r value of 9.51, showed that replacing CH_3_ with Cl (chlorine) had a substantial effect. The substitution of F (fluorine) for CH_3_ had a minor impact, with fluoro pentane having an Fp of −12 °C and r of 4.24 and hexane having an Fp of −22 °C and r of 2 [21]. The operating window for different solvents is discussed in the Supplementary Materials (Figure S1).

### 4.1. Thermal Properties

Several ester solvents that achieve a suitable balance between viscosity and ammonium salt dissolution were also examined. A co-solvent was used to lower the high melting point of ethylene carbonate (EC). However, the modest effect of ammonium salt on lowering the melting temperature of EC rendered it unsuitable as an alternate solvent for EDLC. The addition of methoxy methyl acetate (MMOA) to EC resulted in a considerable reduces the melting point. The EC/MMOA combination did not crystallize down to −78 °C, and the melting point was observed to be −48 °C. The addition of 1 M SBPBF_4_ lowered the melting point of the solvent mixture. The addition of MMOA resulted in the lowest melting temperature, which was close to −55 °C. These findings are summarized in Table 2.

### 4.2. Conductivity Analysis

**Figure 5:**
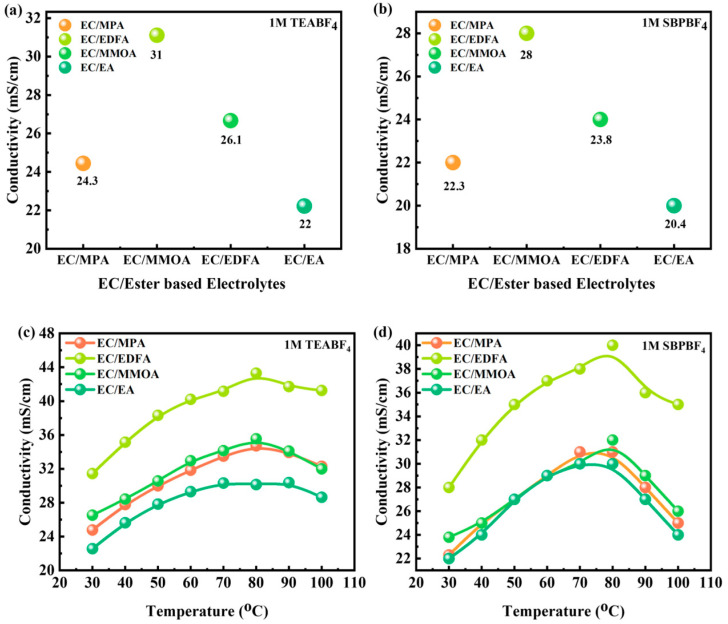
(**a**,**b**) Conductivities of EC/ester-based electrolytes at 30 °C. (**c**,**d**) Conductivity variation with temperature for EC/ester-based electrolytes.

Salt dispersion and viscosity influence the conductivity of electrolytes. Two ammonium salts were tested: TEABF_4_, which is recognized for its low cost and strong electrochemical stability, but has poor solvent solubility, and SBPBF_4_, which has better solvent solubility. At 25 °C, the solubility limits in EC are 2.6 mol/L for TEABF_4_ and 4.2 mol/L for SBPBF_4_. In Figure 5a,b the conductivities of several mixes of the two salts at 30 °C are compared. At 30 °C, the conductivity value of EC/EA with 1 M TEABF_4_ is slightly greater than that of EC/EA with SBPBF_4_, with values of 22 and 20.4 mS/cm, respectively. This discrepancy might be attributed to EC with TEABF_4_ having a lower viscosity (2.9 mPa s at 40 °C) than EC with SBPBF_4_ (3.2 mPa s at 40 °C). The addition of EDFA and MMOA marginally boosts conductivity. Conductivity variation with temperature is shown in Figure 5c,d for 1 M TEABF_4_ and SBPBF_4_ in different solvent mixtures. Because of its increased solubility, SBPBF_4_ allows for a greater quantity of ester co-solvent. Higher temperatures increase conductivity, and the addition of EDFA enhances its conductivity by 1.4 times when compared to EC/EA 1 M TEABF_4_ at 60 °C. Electrolytes solidify at low temperatures; however, with the addition of ester co-solvents, the temperature of solidification drops. In comparison to SBPBF_4_, TEABF_4_ had greater conductivity values at low temperatures.

### 4.3. Viscosity Analysis

The viscosity of electrolytes containing 1 M SBPBF_4_ and 1 M TEABF_4_ was measured at 30 °C to assess the impact of the dissociation of ions and viscosity on the conductivity. The addition of ester-based co-solvents greatly decreased the viscosity of the EC-based electrolyte. The viscosities obtained by adding MMOA were slightly greater. The viscosities of both electrolytes (SBPBF_4_, TEABF_4_) were almost equal, showing that the two electrolytes have similar salt dissociation. The Walden rule was applied to assess the salt dissociation in various electrolytes [21].

(4) ʌ×η=k

**Figure 6:**
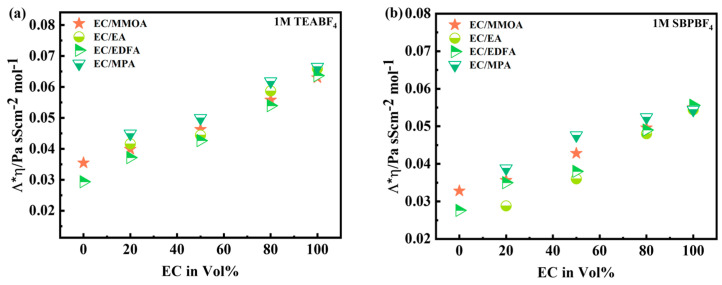
(**a**,**b**) Evolution of Walden product plotted against different Vol% ratios of EC-based electrolytes using 1 M TEABF_4_ and 1 M SBPBF_4_, respectively.

The graph in Figure 6 depicts how ʌ×η fluctuates with electrolyte composition, suggesting variations in salt dissociation with the solvent combination. Because esters have lower dielectric constants (6–8) than $\varepsilon_{r}=90$, they reduce salt dissociation in EC-based electrolytes. When compared to EA ($\varepsilon_{r}=6)$, adding a methoxy or fluorinated group to the ester solvents has a moderate/small influence on the dielectric constant. When the amount of co-solvent is 50% or greater, the influence of co-solvent on salt dissociation becomes considerable. Due to their greater dielectric constant, ester solvents have a smaller influence on dissociation reduction than EA at a 50% proportion. The MMOA had a greater viscosity (h = 0.88 mPa/s) and salt dissociation (ή = 0.42 mPa/s) than EDFA (h = 0.62 mPa/s, ή = 0.41 mPa/s). The product h * L was utilized to compare the salt nature of EC/MMOA-based electrolytes. SBPBF_4_ in EC/MMOA (50/50%) exhibits a little increase in the product h * L in comparison to TEABF_4_ electrolyte due to a minor enhancement in the dissociation of salt. Table 3 presents the viscosities of different electrolytes in 1 M SBPBF_4_ and TEABF_4_. It can be observed that as the volume ratio of MMOA and EDFA increases, the viscosities decrease. However, as discussed earlier, the conductivity increases when incorporating EDFA and MMOA. As a result, a 50/50% ratio is suggested as the optimal blend for achieving the desired conductivity level.

### 4.4. Self-Diffusion Coefficient

Since the viscosity and the conductivity of the SBPBF_4_ and TEABF_4_ electrolytes were almost equivalent, and EDFA is a possible co-solvent, only the EC/EDFA in addition to 1 M TEABF_4_ electrolyte was studied using NMR. The goal of this work was to use a molecular method to expand on the findings acquired using the Walden technique, which is quantitative and macroscopic. Based on their self-diffusion parameters, the Nernst–Einstein equation was used to predict the molar conductivity of the electrolyte, ʌ_NMR_ assuming the dissociation of free ions [21].

(5) $${ʌ}_{NMR}=\frac{N_{A}e^{2}\left(D^{+}+D^{-}\right)}{KT}$$

The cationic and anionic diffusion coefficients of the electrolyte are represented by D^+^ and D^−^, respectively. Other variables in the equation include Avogadro’s number (N_A_), the temperature (T), the charge on ions (e), and the Boltzmann constant (k). The diffusion coefficient for NMR quantifies the mobility of all species in a liquid, including single ions, ion pairs, and larger aggregates. Conductivity measurements, on the other hand, are limited. The dissociation ratio was calculated using the Nernst–Einstein equation. However, molar conductivity derived from the diffusion coefficients of NMR typically exceeds conductivity when compared to experimentally derived using impedance measurement (ʌ_imp_) due to the interaction between the ions. The T^+^ (cationic transference number) was calculated using the dissociation degree, given by the ratio of ʌ_imp_ to ʌ_NMR_, or $\alpha ={ʌ}_{imp}/{ʌ}_{NMR}$. Table 4 summarizes the findings of this study. The addition of EDFA increases ion and solvent mobility (self-diffusion), as demonstrated by a reduction in viscosity. The mobility of the $BF_{4}^{-}$ anion in EC electrolyte is somewhat greater than that of the TEA^+^ cation. TEA^+^ have bare ionic radii of 0.73 nm and $BF_{4}^{-}$ had 0.55 nm, respectively. In EC, observed ion mobility is substantially related to solvated ion sizes, which are relatively comparable. The inclusion of EDFA raises the cationic transference number, with T^+^ rising from 0.43 to 0.48 in the EC/EDFA-based electrolyte. Because the solvation shell is reliant on the solvent characteristics, this rise in T^+^ might be ascribed to stronger $BF_{4}^{-}$ anion coordination solvation in the presence of EDFA or a decreased salt dissociation in an EC/EDFA combination. The second theory is seen to be more feasible, since EDFA has been proven to lower the polarity, increasing the possibility to form more ion pairs. The reported α values imply that not all diffusive species contribute to ionic conduction, and that they can exist as free ions, ionic pairs, or clusters. With the addition of MMOA, the fraction of free ions decreases, as do the α values, which is consistent with its reduced dielectric constant. The NMR results of ion dissociation match well with the qualitative Walden plot data, with a 20% drop in EC/EDFA-based electrolytes in comparison to EC-based electrolytes. EC/EDFA in addition to 1 M ammonium salt electrolytes exhibits an excellent balance between dissociation of ions and viscosity, in contrast to PC with TEABF_4_.

### 4.5. Three-Cell Design

**Figure 7:**
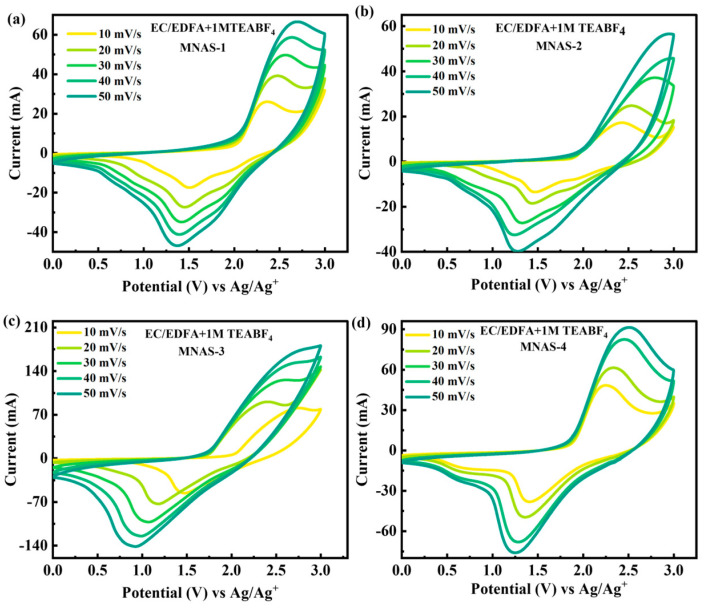
(**a**–**d**) Representation of CV curves for MNAS-1 to MNAS-4 in EC/EDFA + 1 M TEABF_4_ electrolyte.

Because of their high conductivity values, especially at low temperatures, the supercapacitor design was investigated utilizing EC/EDFA + 1 M TEABF_4_ electrolytes. Cyclic voltammetry (CV) studies were performed on four distinct Mg(NbAgS)_x_(SO_4_)_y_ composites, namely MNAS-1, MNAS-2, MNAS-3, and MNAS-4, in an EC/EDFA + 1 M TEABF_4_ electrolyte with a potential window of 0–3 V (Figure 7). The CV curves for all composites displayed redox spikes, suggesting reversible redox processes happening during electrode charging and discharging. The stability of the composites was evaluated at higher scans, and it was discovered that the shape of CV curves remained stable with minor deterioration. MNAS-3 (x = 1 and y = 1) showed a bigger area under the CV curves as compared to other composites, indicating that it had superior capacitance and electrochemical performance. This might be because the Mg(NbAgS)_x_(SO_4_)_y_ combination with x = 1 and y = 1 offered more accessible active sites for ion insertion and extraction, resulting in increased electrochemical performance.

**Figure 8:**
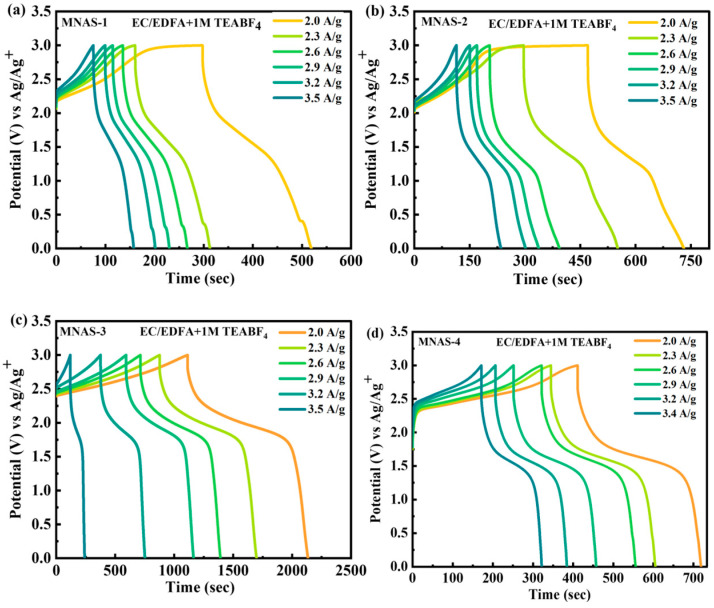
(**a**–**d**) Representation of GCD curves for MNAS-1 to MNAS-4 in EC/EDFA + 1 M TEABF_4_ electrolyte.

The electrochemical performance of Mg(NbAgS)_x_(SO_4_)_y_ composites (MNAS-1, MNAS-2, MNAS-3, and MNAS-4) in EC/MMOA electrolyte was also evaluated by means of galvanostatic charge–discharge (GCD) experiments (Figure 8). The GCD curves exhibited a sloping plateau while charging and a flat plateau during discharging, as expected for intercalation-based materials. MNAS-3 (x = 1 and y = 1) had the largest capacity of all the composites due to its longer discharge time, suggesting higher electrochemical performance. The stability of the composite was further tested at greater current densities of up to 3.5 A/g. These findings imply that the Mg(NbAgS)_x_(SO_4_)_y_ composite in EC/EDFA + 1 M TEABF_4_ electrolyte with x = 1 and y = 1 has the potential to be used as a high-performance cathode material in energy storage devices. Figure S2 represented the electrochemical impedance spectrum (EIS) for MNAS-1 to MNAS-4 composites.

**Figure 9:**
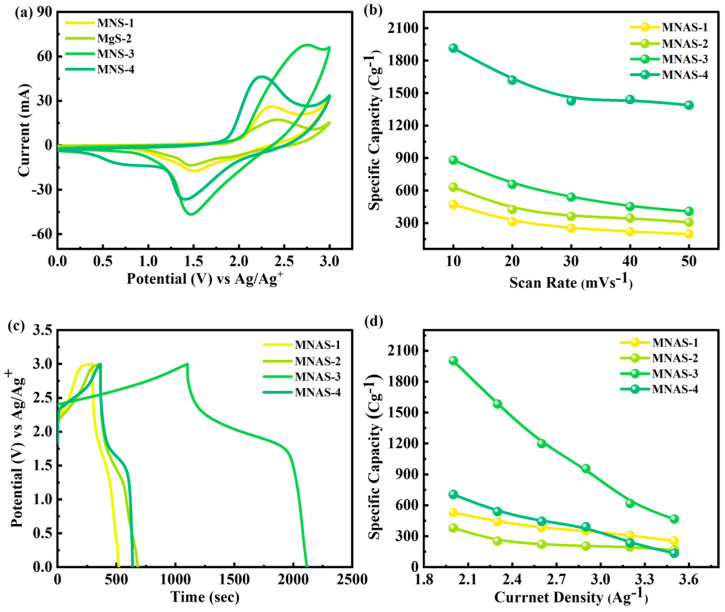
(**a**) Comparison of CV curves for all four composites (MNAS-1 to MNAS-4). (**b**) Specific capacity obtained from CV curves for all four composites (MNAS-1 to MNAS-4). (**c**) Comparison of GCD curves for all four composites (MNAS-1 to MNAS-4). (**d**) Specific capacity obtained from GCD curves for all four composites (MNAS-1 to MNAS-4).

The comparatively drawn CV and GCD curves for MNAS-1 to MNAS-4 through CV and GCD were shown in Figure 9a,c. The specific capacities of MNAS-1, MNAS-2, MNAS-3, and MNAS-4 were determined from the cyclic voltammetry (CV) and galvanostatic charge–discharge (GCD) measurements. The following equation may be used to compute specific capacities [35,36]:

(6) $$Q_{s}=\frac{1}{mv}\int_{V_{i}}^{V_{f}}I\times VdV$$

(7) $$Q_{s}=\frac{I\times t}{m}$$

The parameters used in Equation (6) are active mass (‘*m*’), scan rate (‘*v*’), current (‘*I*’), and operational potential (‘*V*’). In Equation (7), the parameters include current (‘*I*’) discharge duration (‘*t*’), and active mass (‘*m*’). The specific capacities of MNAS-3 were discovered to be extraordinarily high, with values of 1920 C/g (3200 F/g) and 2087 C/g (3478.33 F/g) achieved by CV and GCD, respectively as indicated in Figure 9b,d. This demonstrates the superior electrochemical performance of MNAS-3 as a supercapacitor electrode material. MNAS-3 has a high specific capacity due to the presence of intercalated Mg^2+^ ions and a well-defined layered structure that provides a large surface area and efficient ion transport.

### 4.6. Two-Cell Design

**Figure 10:**
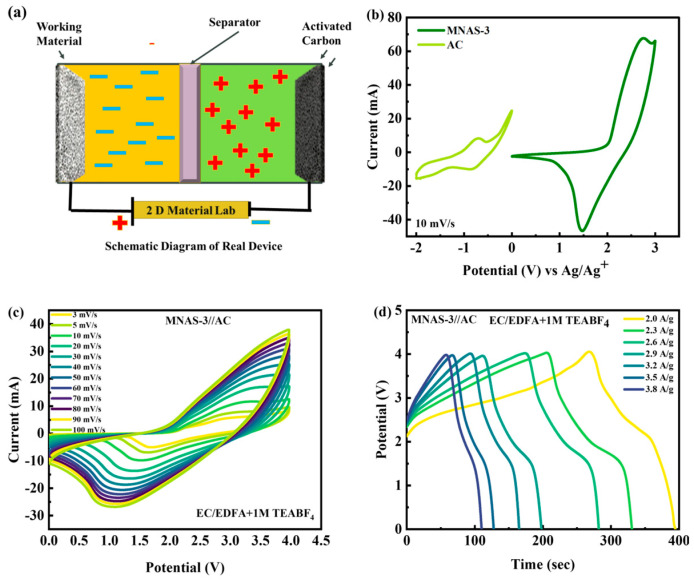
(**a**) Systematic illustration of MNAS-3//AC supercapattery. (**b**) CV comparison of AC and MNAS-3 at 10 mV/s. (**c**) Representation of CV curves for MNAS-3//AC device. (**d**) Representation of GCD curves for MNAS-3//AC device.

Because of its high specific capacity and low impedance values, the MNAS-3 composite with x = 1 and y = 1 was determined to be the most viable choice for supercapattery applications. The systematic representation of MNAS-3//AC supercapattery was represented in Figure 10a. MNAS-3 was integrated with activated carbon (AC) in a hybrid supercapattery device to boost energy storage performance even more. The electrolyte utilized was EC/EDFA + 1 M TEABF_4_, and the operating windows for MNAS-3 and AC were discovered to be 0–3 V and −2–0 V, respectively (Figure 10b). The two cells were separated by a WHATMAN paper, which served as a semipermeable membrane. The combined operational potential (OP) for the MNAS-3//AC device was adjusted to be 0–4 V. The CV curves for the MNAS-3//AC supercapattery were investigated with an operating potential of 0–4 V. The CV curves exhibited rectangular behavior at lower scan speeds, demonstrating the capacitive nature of the system. Redox peaks were identified at greater scan rates, confirming the faradaic character of the system. The stability of the MNAS-3//AC device was further tested at greater scan rates (Figure 10c). The rectangular form of the curves was found to be preserved up to a scan rate of 50 mV/s, beyond which considerable distortion was noticed. However, the redox peaks remained constant up to the greatest scan rate of 100 mV/s, indicating the stability of the faradaic process.

The electrochemical performance of an MNAS-3//AC supercapattery with an OP of 0–4V was evaluated using galvanostatic charge–discharge (GCD) tests (Figure 10d). The GCD curves had a mix of triangular and tiny plateau forms, which is typical of supercapattery behavior. The triangular form shows capacitive behavior, whereas the little plateau indicates the presence of faradaic responses. At greater current densities, the stability of the GCD curves was also studied. The MNAS-3//AC supercapattery demonstrated good stability and generally steady capacitance even at high current densities, suggesting its promise for high-power applications. Figure S3 presents the application of Dunn’s model to the CV curves of the MNAS-3//AC device.

**Figure 11:**
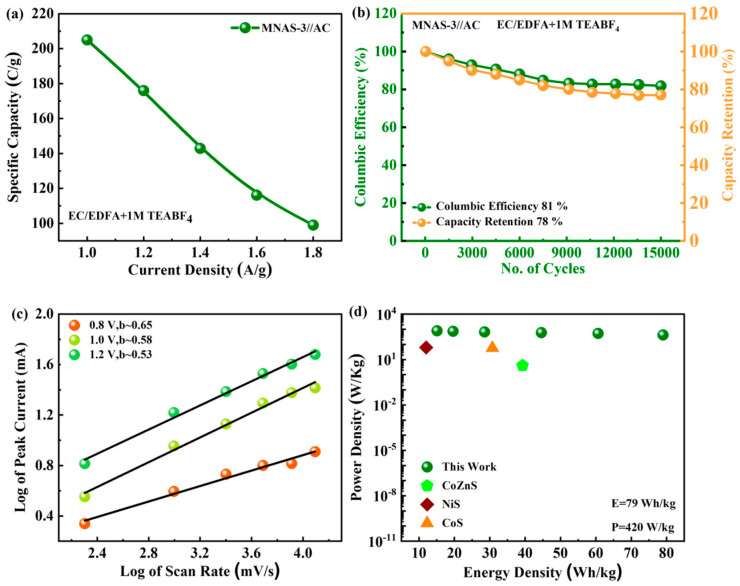
(**a**) Specific capacity calculated for MNAS-3//AC supercapttery through GCD. (**b**) Stability measurement for MNAS-3//AC device after 15,000 cycles. (**c**) b-fitting computed for MNAS-3//AC device at various potentials. (**d**) E_d_ and P_d_ computed for MNAS-3//AC device and compared with past literature.

The specific capacity was also determined from the GCD curves (Figure 11a). The MNAS-3//AC device showed a specific capacity of 208 C/g at a current density of 1.0 A/g. The GCD cycling tests at a current density of 5 A/g were used to assess the stability of the MNAS-3//AC supercapattery (Figure 11b). After 15,000 cycles, the supercapattery demonstrated excellent stability, with 78% capacity retention and 81% Coulombic efficiency. The high capacity retention implies that the electrodes are structurally stable. The charge storage mechanism in the MNAS-3//AC device was determined using b-fitting analysis. To analyze the mechanism, a graph was plotted depicting the logarithm of the scan rate versus the peak current (Figure 11c). The slope of this graph at different potentials fell within the range of 0.5 to 0.8, which corresponds to the theoretical range expected for supercapatteries [33]. The energy and power density of the MNAS-3//AC supercapattery was calculated using the equations [6,7]:

(8) $$E=\frac{Q\times \Delta V}{2\times 3.6}$$

(9) $$P=\frac{E\times 3600}{\Delta t}$$

In the preceding equations, *Q* represents capacity, *V* represents voltage range, *t* represents discharge time, and *E* and *P* indicate energy and power density, respectively. The energy density of the MNAS-3//AC supercapattery was 79 Wh/kg and the power density was 420 W/kg (Figure 11d), indicating that it had a high power output and was able to offer continuous power supply. Table 5 is included in the study to compare our findings to prior research and to confirm the performance of the MNAS-3//AC supercapattery. The results unequivocally display the immense potential of the MNAS-3//AC supercapattery, positioning it as a highly promising solution for applications requiring both high power output and extended operational durations.

## 5. Conclusions

In this study, we investigated the use of ethylene carbonate electrolytes incorporating ester-based solvents for supercapacitors. Furthermore, these ester solvents provide an excellent balance between the dissociation of ions and low viscosity, enhancing the conductivity by up to 31 mS/cm at 30 °C. These findings imply that using ester co-solvents can improve the performance of ethylene carbonate-based electrolytes and help to design high-performance energy storage devices. Additionally, the best electrolyte, EC/EDFA + 1 M TEABF_4_, was used in a three-cell and a two-cell design to investigate the electrochemical performance of Mg(NbAgS)_x_(SO_4_)_y_ composites. The best composite, MNAS-3, showed 2087 C/g capacity, having a current density of 1.0 A/g. The energy density for the MNAS-3//AC device was 79 Wh/kg. The stability measurements were carried out after 15,000 cycles. The developed supercapattery showed an extremely good capacity retention of 78% after 15,000 cycles. This study illuminates a promising future for the utilization of Mg(NbAgS)_x_(SO_4_)_y_ in EC/EDFA + 1 M TEABF4 electrolytes as a formidable contender in next-generation energy storage devices.

## Figures and Tables

**Table 1:** Surface area, pore size, and pore volume computed for MNAS-1 to MNAS-4 composites from BET isotherms.

Materials	SSA (m^2^/g)	Pore Volume (cm^3^/g)	Pore Size (nm)
MNAS-1	33.5	0.023	8
MNAS-2	28.8	0.019	11
MNAS-3	47.3	0.044	15
MNAS-4	39.7	0.037	12

**Table 2:** Crystallization and melting temperature for solvents and electrolytes.

Solution	Melting Point (°C)	Crystallization Temperature (°C)
PC	−49	−56
EC	43	25
EA	−79	--
EC/EA (50/50 wt%)	39	27
EC/EDFA	19	−5
EC/MMOA	−31	−65
EC/MPA	33	20
EC in 1 M SBPBF_4_	38	15
EC/EA in 1 M SBPBF_4_	14	−4
EC/MMOA in 1 M SBPBF_4_	−53	−77
EC/EDFA in 1 M SBPBF_4_	3	−67

**Table 3:** The viscosity of different electrolytes in SBPBF_4_ and TEABF_4_.

Electrolyte Mixture										
Proportion in Vol%	100		80		50		20		0	
	SBPBF_4_/TEABF_4_		SBPBF_4_/TEABF_4_		SBPBF_4_/TEABF_4_		SBPBF_4_/TEABF_4_		SBPBF_4_/TEABF_4_	
EC/MMOA	2.75	2.6	2.48	2.35	2.14	2.07	1.78	1.32	1.64	1.43
EC/EA	2.75	2.6	2.40	2.29	1.80	1.55	1.44	1.26	------	
EC/EDFA	2.75	2.6	2.30	2.22	1.65	1.36	1.19	1.06	1.38	1.20
EC/MPA	2.75	2.6	2.62	2.52	2.38	2.25	1.94	1.61	-----	

**Table 4:** Self-diffusion coefficients for different electrolytes.

Electrolyte	D_TEA+10^6^_ cm/s	$D_{BF_{4}^{-}+10^{6}}$ cm/s	D_EC+10^6^_ cm/s	D_MMOA+10^6^_ cm/s	T^+^	$\sigma_{imp}$ at 40 °C/ms cm^−1^	α
EC	3.98	4.82	5.39	-----	0.43	23.1	0.63
50 EC + 50 EDFA	5.23	6.90	7.33	8.32	0.48	18.7	0.45
80 EC + 20 EDFA	6.04	5.83	6.12	5.12	0.32	20.9	0.54

**Table 5:** Self-diffusion coefficients for different electrolytes.

Electrode Material	Electrolyte	Specific Capacity	Energy Density (Wh/kg)	Power Density (W/kg)
Graphene/SWCNT [54]	Organic	201 F/g	62.8	58.5
Conducting Carbon produced from dead plants [55] CNT-based Supercapacitor [56]	Organic PVDF/PVA	88 F/g 173 F/g	55 -------	------ ------
3D-Graphene electrode [57]	TEABF_4_	23.1 F/g	20	6190
Porous network structured Carbon [58]	Organic	----------	41	67
Ag nanoparticles grown on porous perovskite-type material [59]	1 M KOH	517.5 F/g	21.9	90.3
Vertically aligned CNT [60]	Ionic	75 F/g	27	987
3D porous carbon [61]	Organic	130 F/g	55	2500
**This work**	**EC/EDFA in 1 M TEABF_4_**	**208 C/g**	**79**	**420**

## Data Availability

The data is available upon request.

## Supplementary Materials

The following supporting information can be downloaded at: https://www.mdpi.com/article/10.3390/molecules28124737/s1, Figure S1: CV curves at different solvents to estimate the operating voltage; Figure S2: EIS analysis for MNAS-1 to MNAS-4 composites; Figure S3: Dunn’s model applied on CV curves of MNAS-3//AC device; Table S1: Composition of elements in MNAS-1 to MNAS-4 composites; Table S2: Represent the flashpoint, dielectric constant, and viscosity of the solvents. References [62,63,64] are cited in the supplementary materials.

## Sample Availability

Samples of the compounds are not available from the authors.
