# Supplementary material for: Improving the Energy Storage of Supercapattery Devices through Electrolyte Optimization for Mg(NbAgS)x(SO4)y Electrode Materials
Source: Molecules. 2023 Jun 13;28(12):4737. doi: 10.3390/molecules28124737 (PMC10305711; doi:10.3390/molecules28124737)
Supplement: Supplementary file 1 [file molecules-28-04737-s001.zip › molecules-2433320-supplementary.pdf]

Improving the Energy Storage of Supercapattery Devices through Electrolyte Optimization for Mg(NbAgS)x(SO_4)y Electrode Material

Haseebul Hassan[1], Muhammad Waqas Iqbal[1]*, Sarah Alharthi[2], Mohammed A. Amin[2], Amir Muhammad Afzal[1], Mohd Zahid Ansari[3]*

[1]*Department of Physics, Riphah International University, Campus Lahore, Pakistan*

[2]*Department of Chemistry, College of Science, Taif University, P.O. Box 11099, Taif*

[3]*School of Materials Science and Engineering, Yeungnam University, 280 Daehak-Ro, Gyeongsan, Gyeongbuk 38541, Republic of Korea*

E-mail: waqas.iqbal@riphah.edu.pk, zahid.smr@yu.ac.kr

Supplementary Section:

Different compositions of materials in four composites of $Mg(NbAgS)_x(SO_4)_y$ MNAS-1 to MNAS-4 were represented in Table S1. The physical characteristics of different solvents were represented in Table S2. Chemical changes to esters can increase their flash point, however, this may not impact their redox stability. The platinum electrode was used to investigate the electrochemical windows of different solvents from the cyclic voltammetry measurements. Figures S1(a,b) illustrate voltammograms produced from ester solvents including polar groups. Ethyl acetate and chloromethyl butyrate (ClMB) were discovered to have almost the same oxidation potential. The ethyl difluoro acetate (EDFA) was more resistant to oxidation. The decrease of ClMB occurred at roughly -0.4 V against Ag/Ag+ in the cathodic portion, probably due to the poor stability of the C-Cl bond. This reduction process resulted in the production of an unstable anion radical and the release of Cl^- anion. Furthermore, Propylene Acetate (PA) and

Butyrolactone (BL) displayed discrete voltammograms with varying oxidation and reduction potentials, demonstrating their diverse electrochemical behaviors. The reduction stability of the fluorinated solvent is found to be greater than that of EA, resulting in similar kinetic stability in reduction. This behavior can be ascribed to the higher stability of the C-F bonds when compared to the C-Cl bond. Interestingly, the presence of a methoxy group in the solvent structure does not influence cathodic kinetic stability, as seen in Figure S1. However, the inclusion of methoxy moieties has a significant impact on the anodic potential window, as demonstrated by voltammograms of methoxy methyl acetate (MMOA). The ether function is oxidized in these solvents at roughly 2 V versus Ag/Ag+, which is greater than the oxidation potential of aliphatic ethers like dimethyl ether (1 V vs Ag/Ag+). The voltammograms produced for Propylene Acetate (PA) and Butyrolactone (BL) reveal that the insertion of the ester group in the solvent structure leads to a rise in the flash point. This rise in flashpoints, however, has no negative impact on the redox stability of solvents. The addition of a nitrile group in the ester diminishes its cathodic stability substantially, as indicated by methyl cyanoacetate (MCA) reduction at a potential of roughly 1 V versus Ag/Ag+. The acidic character of the CeH bond, which is positioned close to two electron-withdrawing groups, namely nitrile, and carbonyl, is related to the low electrochemical stability, culminating in the observation of the H^+/H_2 redox system. MCA has a high anodic kinetic stability, even though the form of the curve supports electrode passivation.

Electrochemical impedance spectroscopy (EIS) experiments were performed on four $Mg(NbAgS)_x(SO_4)_y$ composites, namely MNAS-1, MNAS-2, MNAS-3, and MNAS-4. The EIS spectrum were shown in Figure S2. The EIS curves of all four composites had a semi-circle shape, showing that the materials behaved like batteries. The MNAS-3 composite demonstrated a lower equivalent series resistance (ESR) value of 0.96 U, which is advantageous for supercapacitor

applications. The decreased impedance of MNAS-3 can be ascribed to better ion transport capabilities due to the inclusion of both NbS_2 and AgS in the composite, which enables Mg^{2+} ion intercalation/deintercalation. These findings highlight the need of developing materials with improved ion transport characteristics to improve the electrochemical performance of energy storage devices.

The capacitive and diffusive contributions inside the MNAS-3//AC supercapattery at three different scan rates: 3, 50, and 100 mV/s were also measured using Dunn's model approach (Figure S3). Notably, at a scan rate of 3 mV/s, capacitive involvement accounted for 23% of total charge storage, whereas faradic participation accounted for 77% of total charge storage. The capacitive involvement showed a continuously rising trend as the scan rate rose. The increased capacitive involvement can be ascribed to improved ion adsorption and desorption kinetics made possible by the faster scan rate. Surprisingly, at a scan rate of 100 mV/s, capacitive involvement increased to 36%, while diffusive participation decreased to 64%. These findings demonstrate the benefits of increased scan rates in increasing capacitive charge storage, which results in better energy storage performance in the MNAS-3//AC supercapattery.

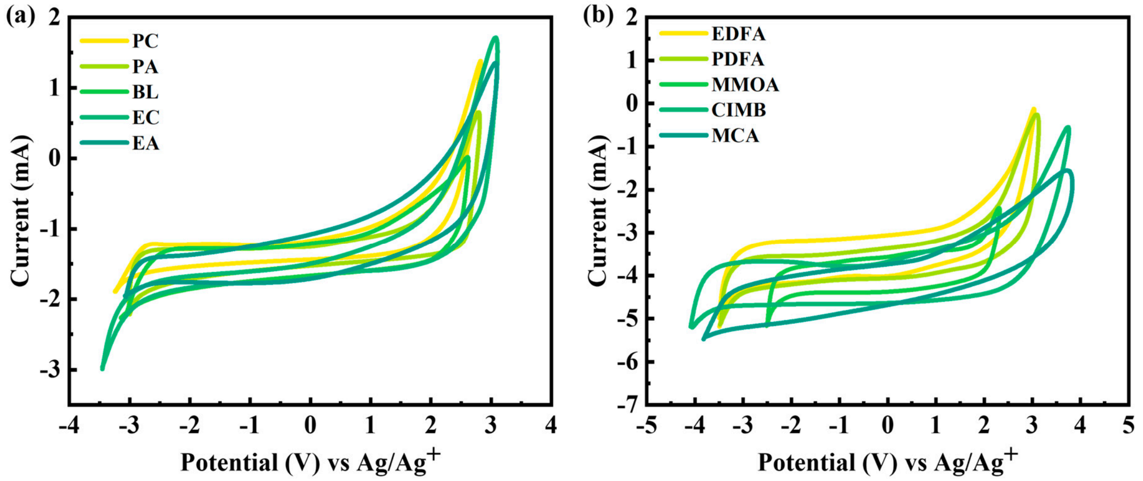

Figure S1. (a-b) Represented the CV curves for different solvents at 10 mV/s to estimate the Operating window.

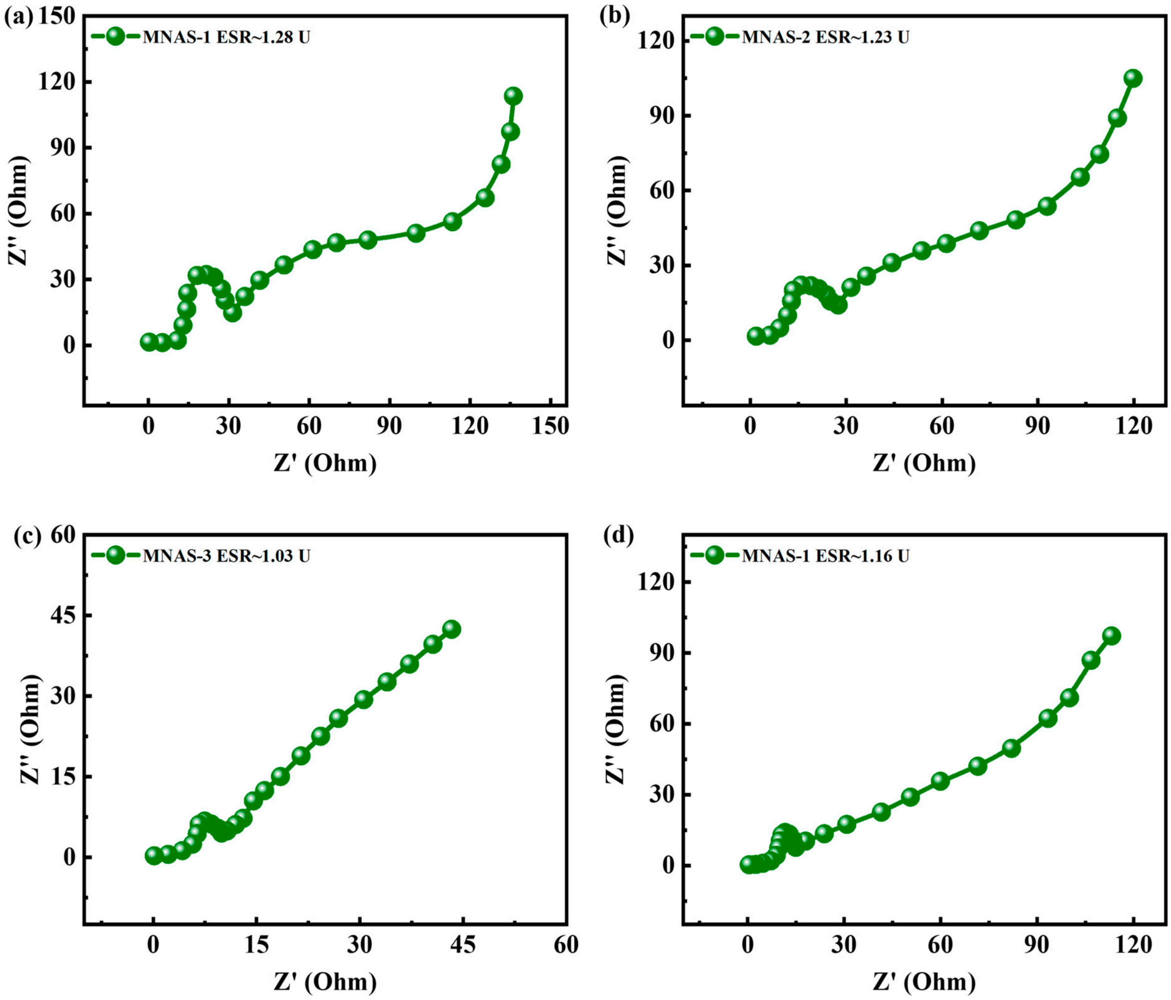

Figure S2. (a-d) EIS spectrum for MNAS-1 to MNAS-4 composites.

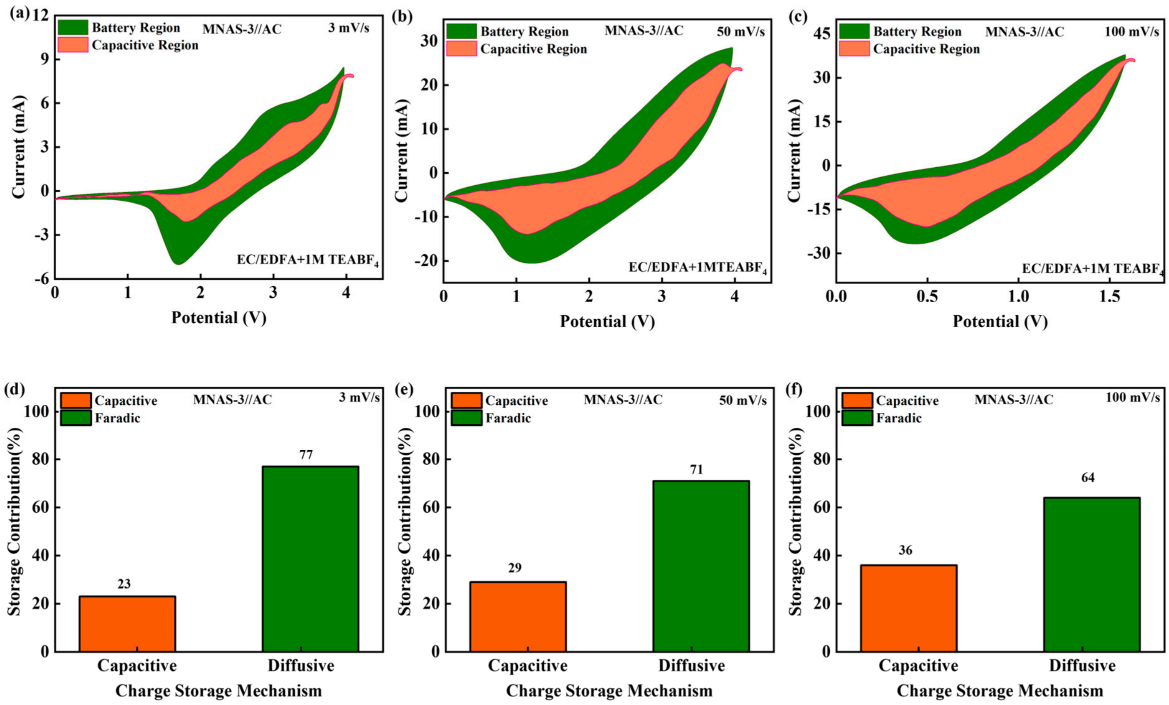

Figure S3. Capacitive and diffusive participation in MNAS-3//AC supercapattery at 3, 50, and 100 mV/s.

Combinations	Mg	NbAgS	SO_4	Compound name
$Mg(NbAgS)_{0.2}(SO_4)_{0.8}$	1	0.2	0.8	MNAS-1
$Mg(NbAgS)_{0.5}(SO_4)_{1.5}$	1	0.5	1.5	MNAS-2
$Mg(NbAgS)_1(SO_4)_1$	1	1	1	MNAS-3
$Mg(NbAgS)_{0.8}(SO_4)_{0.2}$	1	0.8	0.2	MNAS-4

Table S1. Different composition of x and y in $Mg(NbAgS)_x(SO_4)_y$ and their names.

Solvent	Formulla	F_p/C	ή/mPa at 40 °C	ε_r
Propylene Carbonate (PC)	$C_4H_6O_3$	132	2.83	64
Propylene Acetate (PA)	$C_5H_{10}O_2$	25	4.4	6.7
Butyrolactone (BL)	$C_4H_6O_2$	98	1.95	4.13
Ethylene Carbonate (EC)	$C_3H_4O_3$	143	1.96	90
Ethyl Acetate (EA)	$C_4H_8O_2$	-3	0.34	6
Ethyl difluoro acetate (EDFA)	$C_4H_5F_2O_2$	27	0.65	------------
Propylene difluoro acetate (PDFA)	$C_5H_8F_2O_2$	-4	7.12	------------
Methylmethoxyacetate (MMOA)	$C_5H_{10}O_3$	35	0.82	------------
Chloromethylbutyrate (ClMB)	$C_5H_9ClO_2$	55	------------	9.51
Methyl cyanoacetate (MCA)	$C_4H_5NO_2$	43	3.82	28

Table S2. Represented the flashpoint, dielectric constant, and viscosity of solvents. Physical characteristics were obtained from the data sheet form [1-4].

References:

[1] Perricone E, Chamas M, Leprêtre J-C, Judeinstein P, Azais P, Raymundo-Pinero E, et al. Safe and performant electrolytes for supercapacitor. Investigation of esters/carbonate mixtures. Journal of Power Sources. 2013;239:217-24.
[2] Samoc A. Dispersion of refractive properties of solvents: Chloroform, toluene, benzene, and carbon disulfide in ultraviolet, visible, and near-infrared. Journal of Applied Physics. 2003;94:6167-74.
[3] Speight J. Lange's handbook of chemistry: McGraw-Hill Education; 2005.
[4] Maryott AA, Smith ER. Table of dielectric constants of pure liquids: US Government Printing Office; 1951.
